# A cross-sectional study of ocular surface discomfort and corneal nerve dysfunction after paclitaxel treatment for cancer

**DOI:** 10.1038/s41598-021-81398-y

**Published:** 2021-01-19

**Authors:** Jeremy Chung Bo Chiang, David Goldstein, Terry Trinh, Kimberley Au, Susanna B. Park, Arun V. Krishnan, Maria Markoulli

**Affiliations:** 1 School of Optometry and Vision Science, University of New South Wales Sydney, Sydney, Australia; 2 Prince of Wales Clinical School, University of New South Wales Sydney, Sydney, Australia; 3 Brain and Mind Centre, Faculty of Medicine, University of Sydney, Sydney, Australia

**Keywords:** Chemotherapy, Quality of life, Signs and symptoms, Eye manifestations, Peripheral neuropathies

## Abstract

Ocular surface dysfunction is common in patients receiving anti-cancer drug treatment. The effects of paclitaxel, a neurotoxic chemotherapeutic drug, on ocular surface discomfort associated with dry eye disease was investigated. Patients with cancer who had completed paclitaxel treatment between 3 and 24 months prior to assessment (n = 29) and age- and sex-matched healthy control subjects (n = 29) were recruited and assessed with the Ocular Surface Disease Index (OSDI) to measure ocular surface discomfort. In-vivo corneal confocal microscopy was used to evaluate corneal nerve parameters in the right eye. Peripheral neurotoxicity was assessed using patient-reported outcomes and clinical grading scales. The paclitaxel group had significantly worse OSDI total scores compared with controls (Median, Md = 19.3 and Md = 0, *p* = 0.007, respectively). Corneal nerve fiber and inferior whorl lengths were reduced in the paclitaxel group compared with controls (14.2 ± 4.0 and 14.4 ± 4.0 mm/mm^2^ vs. 16.4 ± 4.0 and 16.9 ± 4.9 mm/mm^2^, respectively, *p* = 0.04). When analyzed by presence of peripheral neuropathy, paclitaxel-treated patients with neuropathy showed worse OSDI total scores compared to those without peripheral neuropathy post-treatment (*p* = 0.001) and healthy controls (*p* < 0.001). More severe ocular discomfort and worse visual function was associated with greater peripheral neurotoxicity symptoms (r = 0.60, *p* = 0.001) and neuropathy severity (r = 0.49, *p* = 0.008), respectively. Patients who have been treated with paclitaxel have a higher risk of ocular surface discomfort associated with dry eye disease, particularly those with peripheral neuropathy. Future longitudinal studies should investigate the clinical impact of corneal nerve reduction in dry eye disease.

## Introduction

Dry eye disease is a widespread condition affecting up to 50% of the population^1^. Numerous epidemiological reports show the mental and emotional burden of dry eye disease on societies worldwide with a profound impact on the overall quality of life of affected individuals^2,3^. Such burden of disease is reflected in difficulties experienced by patients in conducting daily activities of living including professional work and driving^4,5^. While risk factors such as the female sex and age have been recognized as risk factors for dry eye disease^1,6^, others such as systemic chemotherapy treatment in cancer are less established. Chemotherapy is one of the most common forms of treatment in cancer. Specific anticancer drugs have been associated with dry eye disease, particularly hormonal agents such as aromatase inhibitors for the treatment of breast cancer^7,8^. However, there is a lack of research for the effects of commonly used cytotoxic anticancer drugs on the ocular surface. While some studies show associations between cytotoxic anticancer drugs with ocular surface dysfunction or dry eye disease^9^, most studies have been limited to case reports or case series. Given the global and chronic impact of dry eye disease on quality of life^10,11^, clinicians should understand the potential long term toxicity of chemotherapy treatment on the ocular surface and the underlying pathophysiological mechanisms.

Certain cytotoxic anticancer drugs are neurotoxic due to their adverse effects on neuronal elements^12^. Specific classes of chemotherapy particularly platinum compounds and taxanes cause nerve damage in patients, principally peripheral neuropathy affecting distal extremities with long-term persistent symptoms of paresthesia or pain in the hands and feet^13,14^. The cornea is the most densely innervated region of the body, about 300–600 times that of the skin^15^, contributing to its sensitivity to a range of stimuli. While research has shown an association between ocular surface dysfunction and sub-basal corneal nerve damage in neuropathic conditions, particularly diabetic peripheral neuropathy^16–18^, evidence is minimal for chemotherapy-induced peripheral neuropathy^19,20^. In-vivo corneal confocal microscopy has been proposed as a useful clinical and research instrument for detecting small nerve fiber changes of the corneal sub-basal nerve plexus in ocular diseases particularly dry eye disease^21^, as well as in the context of diabetic peripheral neuropathy^22,23^ with emerging evidence with neurotoxic chemotherapy from both human and animal studies^19,24^. Images acquired from in-vivo corneal confocal microscopy can be reliably and accurately evaluated for corneal nerve parameters with particular software programs such as ACCMetrics^25,26^. Given the dense innervation of the cornea and the potential association between neurotoxicity and ocular surface dysfunction, the primary aim of this study was to assess ocular surface discomfort associated with dry eye disease in terms of symptomatology and visual function with the validated Ocular Surface Disease Index in patients treated with paclitaxel, one of the most commonly prescribed neurotoxic anticancer drugs used for treatment of breast and gynaecological cancer. A secondary aim was to establish whether ocular surface discomfort is associated with corneal sub-basal nerve changes as detected by in-vivo corneal confocal microscopy, peripheral neuropathic signs and symptoms affecting quality of life.

## Results

**Figure 1:**
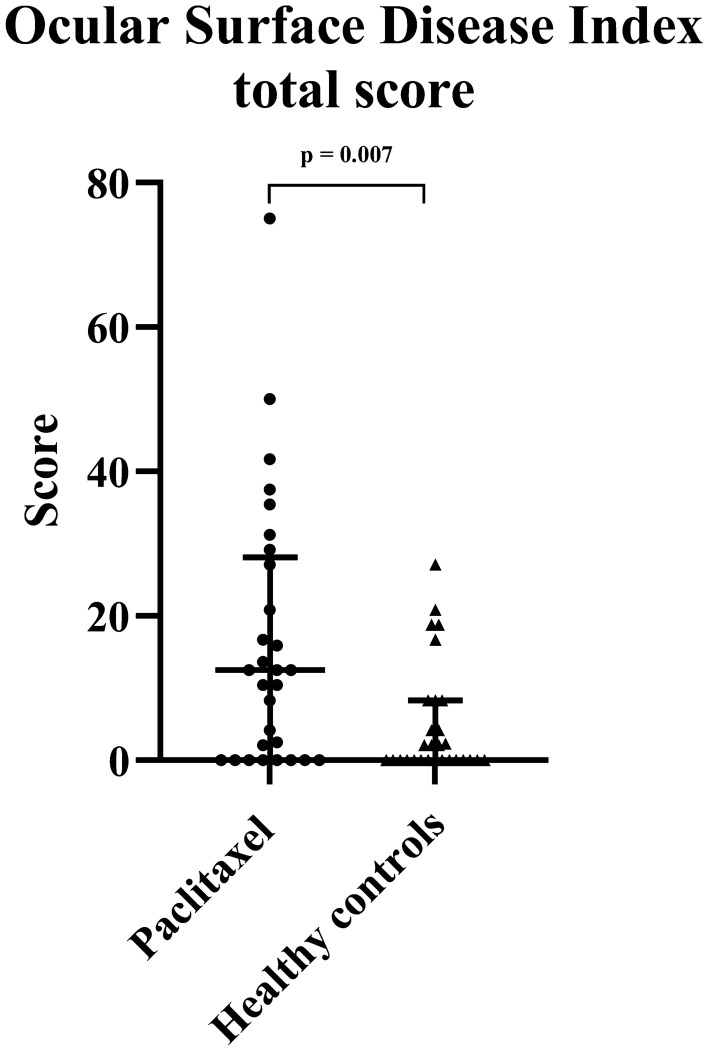
Ocular surface discomfort in paclitaxel-treated patients. Scatterplots of Ocular Surface Disease Index (OSDI) scores for the paclitaxel group and healthy controls. The median and interquartile ranges are also represented.

The prevalence of ocular surface discomfort associated with dry eye disease is presented in Table 1. Paclitaxel-treated patients as a group had higher prevalence of ocular surface discomfort [15 (51.7%) of 29 paclitaxel-treated patients] compared with healthy controls [5 (17.2%) of the 29 healthy controls] (χ^2^ = 7.63, df = 1, *p* = 0.006) (Table 1). The odds ratio for paclitaxel-treated patients experiencing ocular surface discomfort was 5.14 times that of the healthy control group (95% CI 1.54–17.21). The OSDI total score was significantly worse in the paclitaxel group compared to the healthy controls (*p* = 0.007) as shown in Fig. 1 and Table 2, indicating more severe ocular symptomatology in the paclitaxel group. Subcategory scores for OSDI (symptoms) and OSDI (visual function) were also significantly increased in the paclitaxel group compared to healthy controls (*p* = 0.001 and *p* = 0.022, respectively) (Table 2).

**Table 1:** Prevalence of ocular discomfort associated with dry eye disease in all participants and amongst paclitaxel-treated patients with and without neuropathy.

OSDI score and classification of dry eye disease	Participants		Paclitaxel group	
	Paclitaxel group	Healthy controls	With neuropathy	Without neuropathy
≤ 12, none	14 (48.3%)	24 (82.8%)	6 (30%)	8 (88.9%)
> 12, dry eye disease	15 (51.7%)	5 (17.2%)	14 (70%)	1 (11.1%)

OSDI, Ocular Surface Disease Index.

**Table 2:** Clinical characteristics and assessment results of study participants.

Clinical measures	Paclitaxel (n = 29)	Healthy controls (n = 29)	*p*-value
Age, years	53.6 ± 12.7	52.1 ± 14.0	0.27
Gender, female, n (%)	27 (93%)	27 (93%)	
Cumulative dose, mg	1492.79 ± 388.93		
EORTC QLQ-CIPN20	19.3 [3.6–31.6]	0 [0–0]	**< 0.001**
TNSr	3 [1–7]	0 [0–1]	**< 0.001**
**Ocular assessments**			
OSDI score	12.5 [0–28.1]	0 [0–8.3]	**0.007**
OSDI (symptoms)	2.7 [0–8]	0 [0–2]	**0.001**
OSDI (visual function)	0.7 [0–2.7]	0 [0–0.7]	**0.022**
OSDI (environment)	0 [0–4]	0 [0–1.3]	0.202
CNFL (mm/mm^2^)	14.2 ± 4.0	16.4 ± 4.0	**0.04**
IWL (mm/mm^2^)	14.4 ± 4.0	16.9 ± 4.9	**0.04**
CNFD (no/mm^2^)	23.7 ± 7.6	27.8 ± 7.4	0.08
CNBD (no/mm^2^)	34.5 ± 18.8	41.3 ± 23.0	0.25

EORTC QLQ-CIPN20, The European Organization for Research and Treatment of Cancer Quality of Life Questionnaire in Chemotherapy-induced Peripheral Neuropathy Questionnaire scale; TNSr, reduced Total Neuropathy Scale; OSDI, Ocular Surface Disease Index; CNFL, corneal nerve fiber length; IWL, inferior whorl length; CNFD, corneal nerve fiber density; CNBD, corneal nerve branch density.

Data is reported as mean ± standard deviation, or median with interquartile range [Quartile 1–Quartile 2]. The Mann Whitney U test was used to compare the data of the matched groups. Significant results are presented in bold.

**Figure 2:**
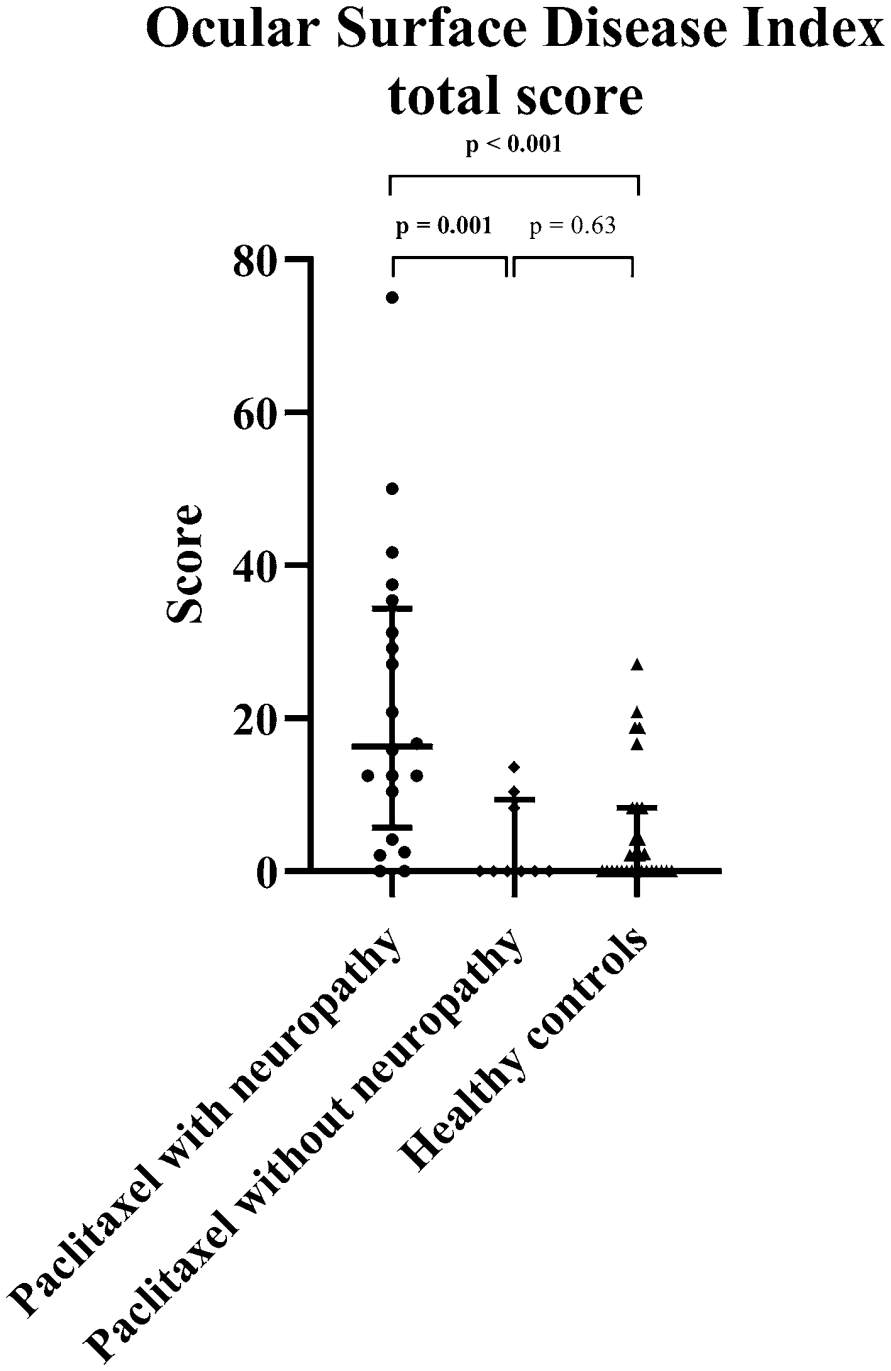
Ocular surface discomfort in the presence of peripheral neuropathy. Scatterplots of Ocular Surface Disease Index (OSDI) scores in paclitaxel-treated patients with persistent peripheral neuropathy, those with no neuropathy and healthy controls. The median and interquartile ranges are also represented. Significant *p*-values are bolded.

When analyzed by presence of peripheral neuropathy, 14 (70%) out of 20 paclitaxel-treated patients with neuropathy were classified as having dry eye syndrome, compared to 1 (11.1%) out of 9 in the subgroup without neuropathy (χ^2^ = 8.62, df = 1, *p* = 0.003, Table 1). The odds ratio for paclitaxel-treated patients with neuropathy experiencing ocular surface discomfort was 18.67 times that of the paclitaxel-treated patients without neuropathy (95% CI 1.89–184.02). Paclitaxel-treated patients with neuropathy showed worse OSDI total scores (*p* = 0.001) compared to those without peripheral neuropathy post-treatment (Table 3 and Fig. 2). Paclitaxel-treated patients with neuropathy also had worse OSDI total scores (*p* < 0.001) and subcategory scores for OSDI (symptoms) and OSDI (visual function) (*p* < 0.001 and *p* = 0.001, respectively) compared to healthy controls (Table 3 and Fig. 2). There was no significant difference in the OSDI total scores between the paclitaxel-treated patients without neuropathy and healthy controls (*p* = 0.63) (Table 3 and Fig. 2).

**Table 3:** Clinical characteristics and assessment results of patients who completed chemotherapy with paclitaxel subcategorized into those with neuropathy and those without neuropathy.

Clinical measures	Participants			*p*-value		
	Healthy controls (n = 29)	Paclitaxel without neuropathy (n = 9)	Paclitaxel with neuropathy (n = 20)	Paclitaxel with neuropathy versus without neuropathy	Paclitaxel with neuropathy versus healthy controls	Paclitaxel without neuropathy versus healthy controls
Age, years	52.1 ± 14.0	42.3 ± 10.1	58.7 ± 10.4	**0.002**	0.13	0.03
Cumulative dose, mg		1644.67 ± 350.28	1443.56 ± 392.59	0.33		
EORTC QLQ-CIPN20	0 [0–0]	1.8 [0–7.2]	21.9 [15.8–37.7]	**< 0.001**	**< 0.001**	0.53
TNSr	0 [0–1]	1 [0–1]	5.5 [3–7.8]	**0.002**	**< 0.001**	0.09
**Ocular assessments**						
OSDI score	0 [0–8.3]	0 [0–9.4]	16.3 [5.7–34.4]	**0.001**	**< 0.001**	0.63
OSDI (symptoms)	0 [0–2]	0 [0–1.3]	5.3 [1.7–9.3]	**< 0.001**	**< 0.001**	0.69
OSDI (visual function)	0 [0–0.7]	0 [0–0.3]	1.7 [0–3.2]	**0.009**	**0.001**	0.75
OSDI (environment)	0 [0–1.3]	0 [0–0.7]	1.3 [0–4]	0.15	0.07	0.81
CNFL (mm/mm^2^)	16.4 ± 4.0	17.2 ± 4.3	12.9 ± 3.0	**0.006**	**0.003**	0.54
IWL (mm/mm^2^)	16.9 ± 4.9	17.4 ± 4.1	13.1 ± 3.2	**0.007**	**0.006**	0.46
CNFD (no/mm^2^)	27.8 ± 7.4	29.2 ± 7.5	21.2 ± 6.3	**0.009**	**0.007**	0.49
CNBD (no/mm^2^)	41.3 ± 23.0	46.8 ± 22.0	28.9 ± 14.7	0.03	0.05	0.44

EORTC QLQ-CIPN20, The European Organization for Research and Treatment of Cancer Quality of Life Questionnaire in Chemotherapy-induced Peripheral Neuropathy Questionnaire scale; TNSr, reduced Total Neuropathy Scale; OSDI, Ocular Surface Disease Index; CNFL, corneal nerve fiber length; IWL, inferior whorl length; CNFD, corneal nerve fiber density; CNBD, corneal nerve branch density.

Data is reported as mean ± standard deviation, or median with interquartile range [Quartile 1–Quartile 2]. Multiple comparisons were conducted with Kruskal–Wallis test with Bonferroni adjustment applied (0.05/3 = 0.017). Significant results (*p* ≤ 0.017) are presented in bold.

**Figure 3:**
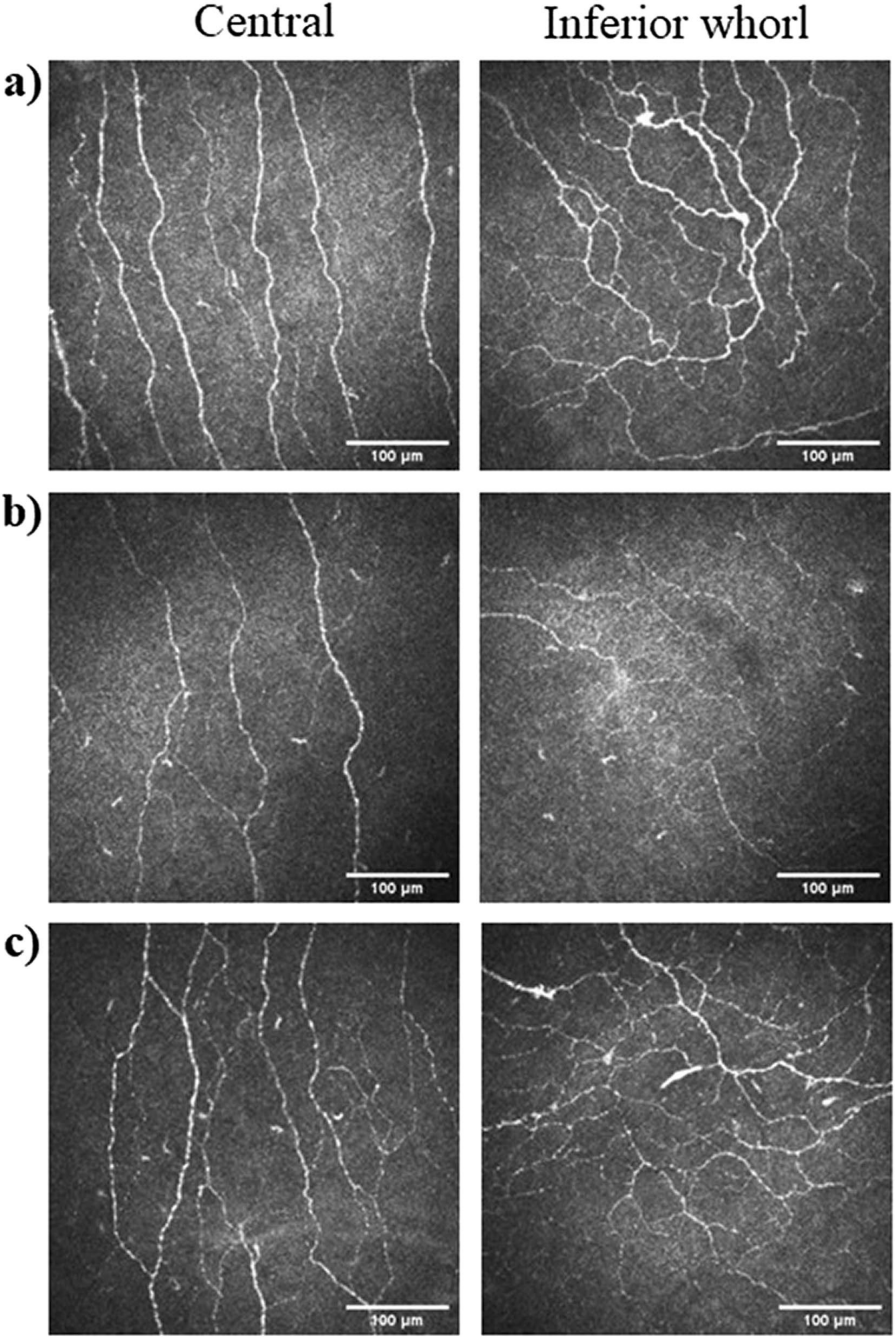
Representative images of corneal sub-basal nerve plexus. Sample images of in-vivo corneal confocal microscopy obtained from the central and inferior whorl region of the cornea in (**a**) a healthy control (age = 61 years), (**b**) paclitaxel-treated patient with peripheral neuropathy (age = 56 years), and (**c**) paclitaxel-treated patient without peripheral neuropathy (age = 58 years).

Representative images of the corneal sub-basal nerve plexus in the central cornea and inferior whorl are shown in Fig. 3. In-vivo corneal confocal microscopy showed significantly reduced CNFL and IWL in the paclitaxel group as a whole compared to healthy controls (*p* = 0.04), while CNFD and CNBD showed no significant differences (Table 2). When the paclitaxel group was subcategorized according to presence of neuropathy, paclitaxel-treated patients with neuropathy showed significantly reduced CNFL compared to those without neuropathy (*p* = 0.006) and healthy controls (*p* = 0.003). IWL was also markedly reduced in paclitaxel-treated patients with neuropathy when compared to those without neuropathy (*p* = 0.007) and healthy controls (*p* = 0.006). Additionally, CNFD was also significantly reduced in paclitaxel-treated patient with neuropathy compared to those without neuropathy (*p* = 0.009) and healthy controls (*p* = 0.007) (Table 3). CNBD was not significantly different between the three groups assessed. CNFL, IWL and CNFD were also similar between paclitaxel-treated patients without neuropathy and healthy controls (Table 3).

**Figure 4:**
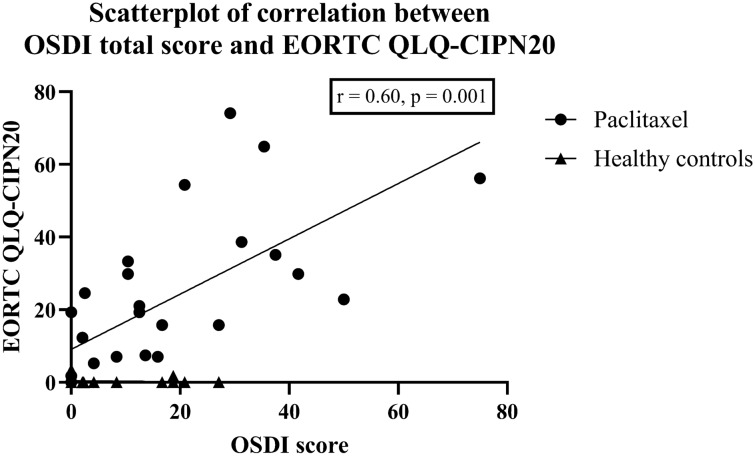
Relationship between ocular surface discomfort and peripheral neuropathy symptoms. Scatterplots of the association between Ocular Surface Disease Index (OSDI) scores with The European Organization for Research and Treatment of Cancer Quality of Life Questionnaire in Chemotherapy-induced Peripheral Neuropathy Questionnaire scale (EORTC QLQ-CIPN20) in paclitaxel-treated patients and healthy controls.

Table 4 shows the association between the OSDI scores with corneal nerve parameters and neurophysiological scores in the paclitaxel group and healthy controls. A worse OSDI total score was associated with more severe neuropathic symptoms as assessed with EORTC QLQ-CIPN20 (r = 0.60, *p* = 0.001) (Fig. 4). Worse subcategory scores for OSDI (symptoms) and OSDI (visual function) were also associated with an increased severity of neuropathic symptoms (r = 0.51, *p* = 0.006 and r = 0.64, *p* < 0.001, respectively). OSDI (visual function) was further correlated with increased neuropathy severity as measured with TNSr (r = 0.49, *p* = 0.008). The OSDI scores did not correlate with CNFL, IWL, CNFD, CNBD or cumulative dose (Table 4).

**Table 4:** Correlation matrix summarizing associations between OSDI scores with neurophysiological measures and corneal nerve parameters within the paclitaxel group after adjusting for age with partial correlation analysis.

	CNFL	IWL	CNFD	CNBD	EORTC QLQ-CIPN20	TNSr	Cumulative dose
**Paclitaxel**							
OSDI score	− 0.03 (*p* = 0.81)	− 0.18 (*p* = 0.18)	0.02 (0.91)	− 0.07 (0.74)	**0.60** (***p* = 0.001**)	0.34 (*p* = 0.07)	0.15 (*p* = 0.46)
OSDI (symptoms)	− 0.08 (*p* = 0.69)	− 0.38 (*p* = 0.05)	− 0.14 (0.49)	− 0.11 (0.57)	**0.51 (* p* = 0.006**)	0.24 (*p* = 0.23)	0.16 (*p* = 0.42)
OSDI (visual function)	− 0.08 (*p* = 0.70)	− 0.35 (*p* = 0.07)	− 0.01 (0.95)	− 0.11 (0.59)	**0.64 (*p*** ** < 0.001)**	**0.49 (***p* ** = 0.008)**	0.17 (*p* = 0.39)
OSDI (environment)	0.23 (*p* = 0.25)	0.08 (*p* = 0.70)	0.36 (0.06)	0.14 (0.47)	0.34 (*p* = 0.08)	0.09 (*p* = 0.66)	0.11 (*p* = 0.57)
**Healthy controls**							
OSDI score	0.25 (*p* = 0.20)	0.17 (*p* = 0.38)	0.19 (0.34)	0.05 (0.79)	− 0.05 (*p* = 0.79)	− 0.10 (*p* = 0.60)	
OSDI (symptoms)	0.23 (*p* = 0.24)	0.13 (*p* = 0.52)	0.17 (0.38)	0.06 (0.77)	− 0.11 (*p* = 0.59)	− 0.01 (*p* = 0.94)	
OSDI (visual function)	0.28 (*p* = 0.15)	0.24 (*p* = 0.23)	0.18 (0.36)	0.16 (0.42)	0.07 (*p* = 0.71)	− 0.10 (*p* = 0.63)	
OSDI (environment)	0.12 (*p* = 0.55)	0*.*07 (*p* = 0.71)	0.11 (0.58)	− 0.04 (0.82)	− 0.06 (*p* = 0.77)	− 0.13 (*p* = 0.50)	

Data is reported as r (*p*-value), with statistically significant correlations highlighted in bold.

OSDI, Ocular Surface Disease Index; EORTC QLQ-CIPN20, The European Organization for Research and Treatment of Cancer Quality of Life Questionnaire in Chemotherapy-induced Peripheral Neuropathy Questionnaire scale; TNSr, reduced Total Neuropathy Scale; CNFL, corneal nerve fiber length; IWL, inferior whorl length; CNFD, corneal nerve fiber density; CNBD, corneal nerve branch density. Bonferroni adjustment was applied (0.05/6 = 0.0083). Significant results (*p* ≤ 0.0083) are presented in bold.

## Discussion

The present study explored ocular surface discomfort associated with dry eye disease in patients treated with paclitaxel. This study showed that patients treated with paclitaxel had a higher prevalence of ocular surface discomfort and more severe ocular symptoms and loss of visual function compared with healthy controls. In particular, paclitaxel-treated patients who developed neuropathy had a higher risk of experiencing ocular surface discomfort, while those without neuropathy had a lower risk.

Of clinical importance, more severe ocular surface discomfort and visual function was associated with worse peripheral neuropathic symptoms affecting daily activities of living and peripheral neuropathy severity, respectively, in paclitaxel-treated patients. There may be a contribution from doxorubicin, cyclophosphamide or carboplatin, drugs involved in the treatment regimen, towards the ocular surface discomfort observed. However, it is unlikely that these drugs are major factors, as the current data showed that patients with peripheral neuropathy, which is primarily caused by paclitaxel, have more severe ocular surface discomfort compared to those without neuropathy. The strong association between ocular surface discomfort and peripheral neuropathy symptoms as well as loss of visual function with peripheral neuropathy severity further suggested that neurotoxicity associated with paclitaxel was more likely to be the main culprit.

The lack of association between ocular surface discomfort and corneal nerve reduction reflects the recurring theme in dry eye disease diagnosis where there is often a lack of correlation between signs and symptoms^27^. This adds to the conundrum in understanding dry eye disease pathophysiology and symptomatology. The cornea is known to be the most densely innervated part of the human body with unmyelinated small nerve fibers, contributing to its high sensitivity to various stimuli^15^. Reduction in corneal nerve parameters was evident in paclitaxel-treated patients, particularly those with peripheral neuropathy. Although the corneal nerve parameters measured in the current study showed no link with ocular surface discomfort, paclitaxel treatment has previously been shown to cause severe reduction in intraepidermal nerve fibers expressing the neuropeptide substance P, contributing to neuropathic symptoms experienced by rats^28^. As a substantial percentage of the sensory small nerve fibers innervating the ocular surface, particularly the cornea, also express substance P^29^, this may be the link between paclitaxel neurotoxicity and the ocular surface discomfort experienced by treated patients, and will be the subject of evaluation in future studies.

The limitations of the current study include its cross-sectional design and limited number of paclitaxel-treated patients with and without neuropathy. There appears to be a strong association in patients with paclitaxel-induced peripheral neuropathy and ocular surface discomfort. However, only future longitudinal studies which involve a prospective cohort, where patients are followed prior to, during and after neurotoxic chemotherapy treatment, will definitively establish the natural history of changes in ocular surface dysfunction and the true impact of paclitaxel and other neurotoxic drugs. The study also cannot exclude an even greater effect during or immediately after cessation of paclitaxel chemotherapy, however, even at a delayed time point, there appear to be clinically significant ocular surface discomfort and peripheral neuropathic symptoms. While the age of the patients with neuropathy in the current study is greater than those without neuropathy, the reduction is unlikely to be due solely to an age effect as the observed difference exceeds the normal aging decrease shown by a multinational normative dataset (− 0.06 mm/mm^2^ per year in CNFL^30^; − 0.171 no/mm^2^ per year in CNFD^31^). Objective assessments of ocular surface integrity including non-invasive tear break up time and vital staining with fluorescein or lissamine green will also be valuable to further investigate the association between signs and symptoms of ocular surface dysfunction. The current study mainly focused on the ocular surface discomfort symptoms that are affecting the quality of life of patients and corneal nerve fiber dysfunction. Future studies should differentially diagnose dry eye syndrome from ocular neuropathic pain clinically.

In conclusion, patients with cancer who have been treated with paclitaxel, a neurotoxic chemotherapy treatment, have a higher risk of ocular surface discomfort associated with dry eye disease compared to age- and sex-matched healthy controls, more so in the presence of peripheral neuropathy. Eye care clinicians should be aware of the ocular surface discomfort in cancer patients treated with neurotoxic chemotherapy, while medical oncologists should be cognizant of such effects in patients who have completed treatment particularly those with persistent peripheral neuropathic symptoms. Future longitudinal studies incorporating objective ocular surface measures with prospective cohorts are warranted to further investigate these associations.

## Methods

### Study design

This was a prospective, observational cross-sectional study which involved a single study visit, approved by the South Eastern Sydney Area Health Service Human Research Ethics Committee. Participants provided written informed consent in accordance with the Declaration of Helsinki. All methods were conducted in accordance with relevant guidelines and regulations.

### Patient selection

Twenty-nine participants with breast, ovarian or endometrial cancer who had completed paclitaxel treatment (26 treated with paclitaxel + doxorubicin + cyclophosphamide (AC regimen) and 3 treated with paclitaxel + carboplatin (TC regimen)) between 3 and 24 months ago were recruited from the Department of Medical Oncology, Prince of Wales Hospital (Sydney, Australia). Twenty-nine age- and sex-matched healthy control subjects were also recruited through contact in the local health district. Sample size calculations were based on the minimally important difference for OSDI total score (the primary measure of the current study) of 10 for mild to moderate dry eye disease at a standard deviation for change of 13. At 80% power and 95% confidence, this gives a sample size of 22 participants^32^.

Participants were excluded if they had a history of any other medical disorder known to cause neuropathy including pre-diabetes, diabetes and chronic kidney disease, were pregnant or lactating, had a history of ocular trauma, refractive surgery or contact lens wear, or had any active ocular disease such as iritis, corneal edema, corneal dystrophies or herpes simplex keratitis. Medication use which could potentially cause dry eye syndrome such as isotretinoin or oral contraceptives were also included in the exclusion criteria. Patients with cancer treated with hormonal agents including tamoxifen and aromatase inhibitors at the time of assessment were also excluded as these medications have been shown to cause ocular surface dysfunction^7,8^.

### Procedures

All participants underwent clinical assessments which involved a series of subjective questionnaires and objective neurological assessment. As part of a clinical suite of neurological tests, peripheral neuropathic symptoms and effect on daily activities of living were assessed with The European Organization for Research and Treatment of Cancer (EORTC) Quality of Life Questionnaire in Chemotherapy-induced Peripheral Neuropathy (QLQ-CIPN20)^33^. These include numbness, tingling, shooting or burning pain and cramps in the distal extremities. The reduced version of the Total Neuropathy Scale (TNSr, Johns Hopkins University) was used to grade CIPN with symptomatology, clinical and neurophysiological measures across 8 domains from a scale of 0–4 (sensory symptoms, weakness symptoms, pin prick sensibility, vibration threshold, strength, deep tendon reflexes, sensory sural nerve and motor tibial nerve conduction studies)^34,35^. The total score for each participant was gathered (0–32), with larger scores reflecting greater neuropathy. Chemotherapy data including cumulative dose were collated.

Participants were assessed with the Ocular Surface Disease Index (OSDI), a 12-item questionnaire developed to assess the severity of dry eyes in relation to three subcategories: severity of ocular symptoms including light sensitivity, grittiness, pain or soreness [OSDI (symptoms)], visual function issues related to blurred or poor vision, reading, driving at night, working with a computer or bank machine, and watching television [OSDI (visual function)] and susceptibility to environmental triggers including windy conditions, places of low humidity and air conditioned areas [OSDI (environment)]^36^. Scores were calculated with the following formula:

$$Score=\frac{\left(Sum\, of\, scores\, for\, all\, questions\, answered\right)\times 100}{\left(Total\, number\, of\, questions\, answered\right)\times 4}$$

The total score ranges from 0 to 100, with a total score > 12 indicating presence of dry eye syndrome^32^. Subcategory scores are calculated similarly, considering only the questions from each subcategory to generate its own score and hence the subcategory scores also have a range of 0–100^36^.

In-vivo corneal confocal microscopy was performed with a laser scanning confocal microscope consisting of a diode laser with a wavelength of 670 µm (Heidelberg Retinal Tomograph III with Rostock Corneal module; Heidelberg Engineering GmbH, Heidelberg Germany) on participants after the cornea of the right eye had been anaesthetized with 1 drop of sterile 0.4% benoxinate hydrochloride (oxybuprocaine hydrochloride). Eight images best representing the central cornea with less than 20% overlap between images, and five images representing the inferior whorl region were identified^31,37,38^. Each image requires an acquisition time of 0.024 s, and consists of 384 × 384 pixels which corresponds to an area of 400 µm × 400 µm^39^. An established automated image analysis software capable of fully automated nerve fiber detection and tracing (ACCMetrics, The University of Manchester Intellectual Property UMIP, Manchester, United Kingdom)^25,26^ was used to assess corneal nerve fiber length (CNFL, mm/mm^2^), the total length of all nerve fibers in the central cornea (main trunks and branches) and inferior whorl length (IWL, mm/mm^2^), the total length of all nerve fibers and branches in the inferior whorl region, both of which have been shown to be the most reliable and sensitive measures of peripheral neuropathy in the diabetic context^40,41^ and have shown involvement in dry eye diseases^21^. The inferior whorl region is approximately 1–2 mm inferonasal to the central apex of the cornea and is a distinct landmark of the corneal sub-basal nerve plexus signifying the most distal portion of corneal nerves^42^ (Fig. 3). Additional corneal nerve parameters including corneal nerve fiber density (CNFD, no/mm^2^) and corneal nerve branch density (CNBD, no/mm^2^) were also assessed due to their involvement in dry eye disease^21^ and diabetic peripheral neuropathy^23^. The evaluation of corneal nerves with in-vivo corneal confocal microscopy combined with ACCMetrics, the well-validated fully automated nerve tracing and analysis software, has been shown to have high intraobserver and interobserver reliability^43^, with widespread clinical research usage in patients with dry eye disease^21^ and those with peripheral neuropathy particularly in diabetes^19,40^.

### Statistical analysis

Data analysis was performed with IBM SPSS Statistics (version 25). Chi-squared test of independence was used for comparing the prevalence rates of ocular surface discomfort associated with dry eye disease between participants. The Mann Whitney U test was used for statistical analysis to compare the paclitaxel group with age- and sex-matched healthy controls. A *p* ≤ 0.05 was considered significant. A sub-analysis which stratified participants to grade 0 or no neuropathy and those with neuropathy (grade ≥ 1) according to TNSr (Scores for grade 0: 0–1; grade 1: 2–8; grade 2: 9–16; grade 3: 17–24; grade 4: 25–32) was also performed to investigate the difference in ocular symptomatology between the two groups and healthy controls. The Kruskal–Wallis test was used to compare the data, and to control for type 1 errors, Bonferroni adjustment was applied to multiple comparisons amongst the three groups in this analysis to assess for statistical significance. An adjusted *p*-value (0.05/3) was used to assess for significance. Partial correlation analysis was performed to investigate the association between OSDI with corneal nerve parameters, EORTC QLQ-CIPN20 and TNSr scores accounting for age by adding participant age into the correlation analysis for each of the paclitaxel and healthy controls groups. Age has been shown to be a factor in dry eye syndrome^1^, corneal nerve reduction^31^ and peripheral neuropathic status^44^. Bonferroni adjustment was applied to the correlation analysis which evaluated the association of the scores of OSDI with four corneal nerve parameters and two neurophysiological measures in paclitaxel group and healthy controls with an adjusted *p*-value (0.05/6) to assess for significance. The association between cumulative dose of paclitaxel and OSDI scores were also evaluated.

## Data Availability

The datasets generated and analysed during the study are available from the corresponding author on reasonable request.
